# Supplementary material for: Rare Phytocannabinoids Exert Anti-Inflammatory Effects on Human Keratinocytes via the Endocannabinoid System and MAPK Signaling Pathway
Source: Int J Mol Sci. 2023 Feb 1;24(3):2721. doi: 10.3390/ijms24032721 (PMC9917187; doi:10.3390/ijms24032721)
Supplement: Supplementary file 1 [file ijms-24-02721-s001.zip › ijms-2175756-supplementary.pdf]

Rare phytocannabinoids exert anti-inflammatory effects on human keratinocytes via the endocannabinoid system and MAPK signalling pathway

Daniel Tortolani[1,2#], Camilla Di Meo[3#], Sara Standoli[3], Francesca Ciaramellano[1], Salam Kadhim[4], Eric Hsu[4], Cinzia Rapino[1*] and Mauro Maccarrone[2,5*]

[1]*Department of Veterinary Medicine, University of Teramo, Teramo, 64100, Italy*

[2]*European Center for Brain Research (CERC)/Santa Lucia Foundation IRCCS, Rome, 00143, Italy*

[3]*Department of Bioscience and Technology for Food Agriculture and Environment, University of Teramo, Teramo, 64100, Italy*

[4] *InMed Pharmaceuticals Inc., Vancouver BC, V6C 1B4, Canada*

[5] *Department of Biotechnological and Applied Clinical Sciences, University of L'Aquila, 67100 L'Aquila, Italy*

[#] *Equally first authors.*

* *Correspondence: crapino@unite.it (C.R.); mauro.maccarrone@univaq.it (M.M.)*

Supplementary materials

Dose-response curves of tested interleukins and representative images of phospho-kinase arrays

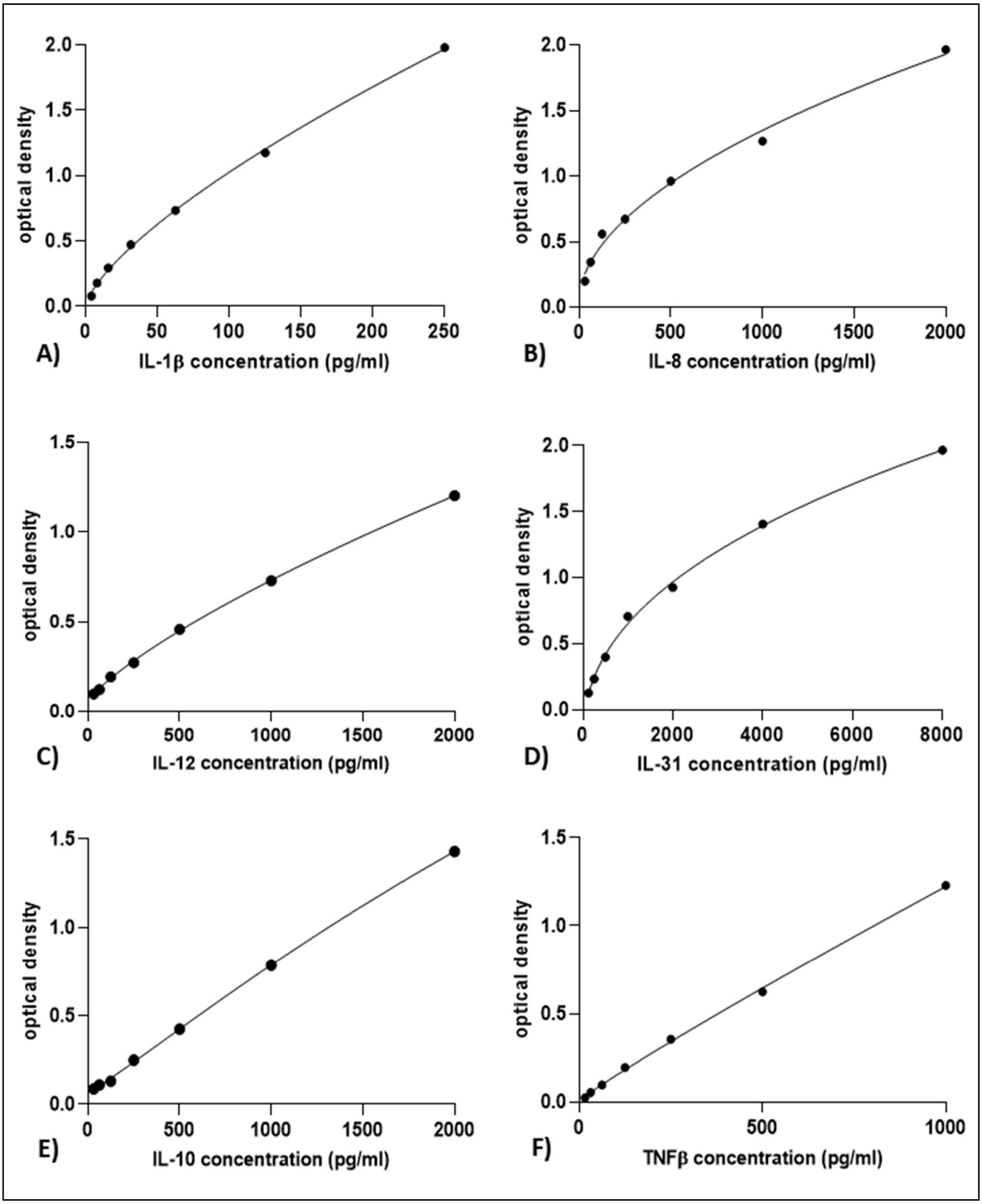

Figure S1. Dose-response curves of tested interleukins (pg/ml). A) IL-1β; B) IL-8; C) IL-12; D) IL-31; E) IL-10; and F) TNFβ.

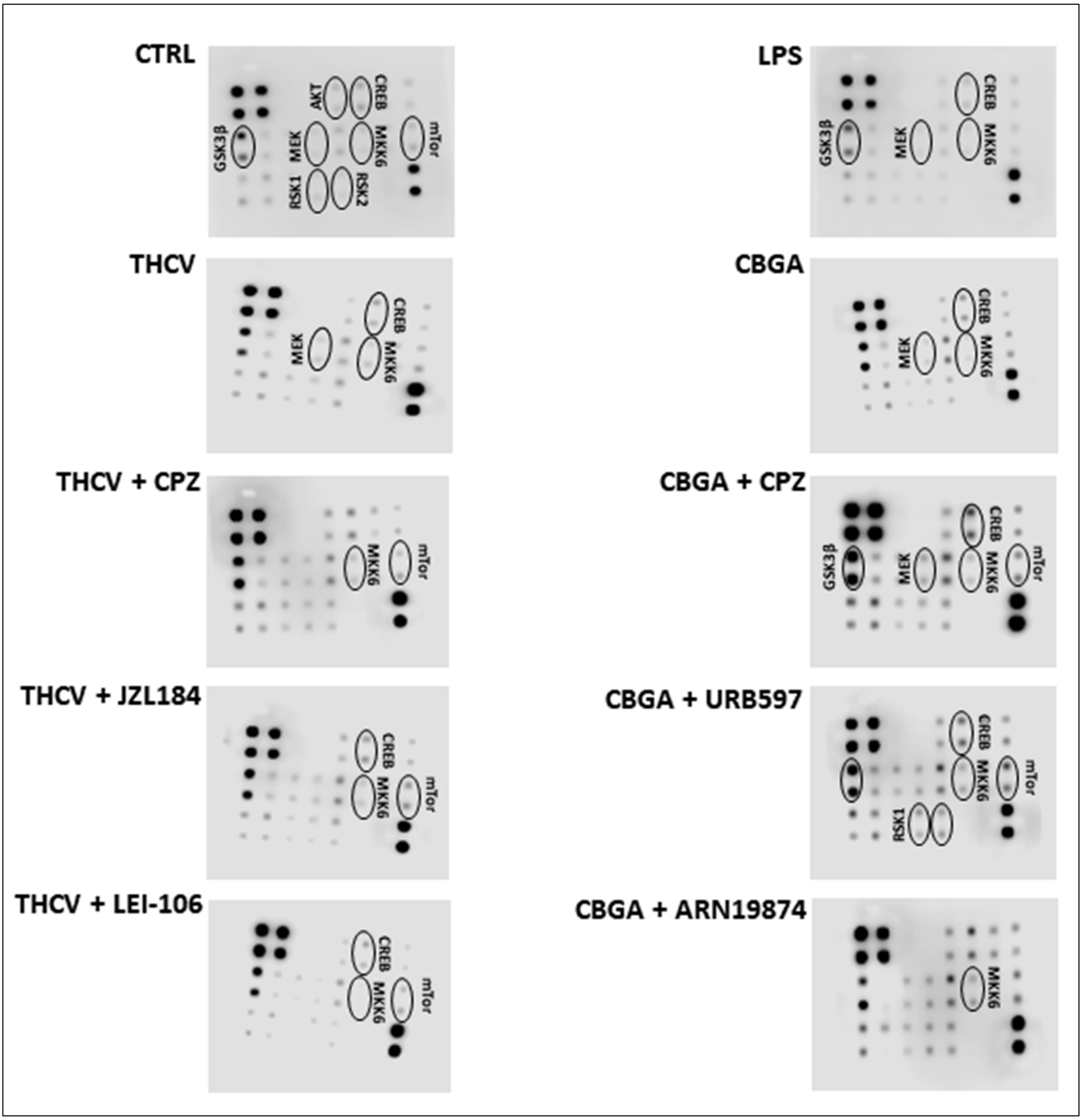

Figure S2. Representative images of phospho-kinase arrays for each treatment, captured by C-DiGit blot scanner. Each membrane detects the following 17 MAPKs: serine/threonine kinase 1 (AKT); cyclic adenosine monophosphate (cAMP) response element-binding protein (CREB); Glycogen synthase kinase 3 α (GSK3α) and -β (GSK3β); c-Jun N-terminal kinase (JNK); extracellular signal-regulated kinase (ERK1); mitogen-activated protein kinase (MEK1); Mitogen-activated protein kinase kinase 3 (MKK3) and 6 (MKK6); mitogen- and stress-activated protein kinase 2 (MSK2); Heat shock protein 27 (HSP27); mammalian target of rapamycin (mTor); p38 mitogen-activated protein kinase (p38); tumour suppressor protein (p53); p70 ribosomal S6 kinase (P70S6k), Ribosomal S6 kinase 1 (RSK1) and 2 (RSK2) (each spotted in duplicate). The pairs of dots in the upper left corner and the two dots in the lower right corner are positive controls. Black circles denote the 8 proteins(AKT, CREB, GSK3β, MEK, MKK6, mTor, RSK1 and RSK2) with significant variations under different experimental conditions.

Table S1. Representative membrane with distribution of antibodies used for MAPK array. POS = Positive Control Spot, used for normalization. NEG = Negative Control Spot, used to measure the baseline.

Each antibody is spotted in duplicate vertically		A	B	C	D	E	F	G	H
	1<br>2	POS	POS	NEG	NEG	Akt<br>(P-S473)	CREB<br>(P-S133)	ERK1<br>(P-T202/Y204)<br>ERK2<br>(P-Y185/Y187)	GSK3a<br>(P-S21)
	3<br>4	GSK3b<br>(P-S9)	HSP27<br>(P-S82)	JNK<br>(P-T183)	MEK<br>(P-S217/221)	MKK3<br>(P-S189)	MKK6<br>(P-S207)	MSK2<br>(P-S360)	mTOR<br>(P-S2448)
	5<br>6	p38<br>(P-T180/Y182)	P53<br>(P-S15)	P70S6K<br>(P-T421/S424)	RSK1<br>(P-S380)	RSK2<br>(P-S386)	NEG	NEG	POS
